# The biogenic amines putrescine and cadaverine show *in vitro* cytotoxicity at concentrations that can be found in foods

**DOI:** 10.1038/s41598-018-36239-w

**Published:** 2019-01-15

**Authors:** Beatriz del Rio, Begoña Redruello, Daniel M. Linares, Victor Ladero, Patricia Ruas-Madiedo, Maria Fernandez, M. Cruz Martin, Miguel A. Alvarez

**Affiliations:** Dairy Research Institute, IPLA-CSIC, Paseo Rio Linares s/n, 33300 Villaviciosa, Spain

## Abstract

Putrescine and cadaverine are among the most common biogenic amines (BA) in foods, but it is advisable that their accumulation be avoided. Present knowledge about their toxicity is, however, limited; further research is needed if qualitative and quantitative risk assessments for foods are to be conducted. The present work describes a real-time analysis of the cytotoxicity of putrescine and cadaverine on intestinal cell cultures. Both BA were cytotoxic at concentrations found in BA-rich foods, although the cytotoxicity threshold for cadaverine was twice that of putrescine. Their mode of cytotoxic action was similar, with both BA causing cell necrosis; they did not induce apoptosis. The present results may help in establishing legal limits for both putrescine and cadaverine in food.

## Introduction

Biogenic amines (BA) are naturally occurring nitrogenous compounds synthesised by plants, animals and microorganisms, mainly through the decarboxylation of amino acids. BA can accumulate at high concentrations in foods, largely due to the metabolic activity of microorganisms with amino acid decarboxylase activity^1–4^. Foods likely to contain high BA concentrations are fish, fish products and fermented foodstuffs (meat, dairy, some vegetables, beers and wines)^5–8^. Although BA have important biological functions in humans and are endogenously synthesised, the ingestion of food with high concentrations of BA can provoke serious toxicological reactions^6,8–12^.

According to their chemical structure and number of amine groups, the BA putrescine and cadaverine are aliphatic diamines. The European Food Safety Authority (EFSA) has declared them among the most common BA found in foods^12^. Putrescine can accumulate at high concentrations in dairy fermented products such as cheese (up to 1560 mg/kg), fermented sausages (up to 1550 mg/kg), fish sauces (up to 1220 mg/kg), fermented vegetables (up to 549 mg/kg), and fish and fish products (up to 337 mg/kg)^12^. Cadaverine can accumulate at high concentrations in cheese (up to 3170 mg/kg), fish and fish products (up to 1690 mg/kg), fermented sausages (up to 1250 mg/kg) and fish sauces (up to 1150 mg/kg)^12^. Certainly, they are among the most abundant BA found in cheeses, along with tyramine and histamine^8,11,13^ (considered by the EFSA as the most toxic of all BA^12^).

The information about the toxicity of putrescine and cadaverine is scarce, no human dose-response data are available and only one animal study has been published in which a non-observed adverse effect level (NOAEL) of 2000 ppm (180 mg/kg body weight/day) was established in Wistar rats^14^. Although the pharmacological activities of putrescine and cadaverine seem less potent than those of histamine and tyramine^12^, the consumption of these vasoactive BA has been related to acute un-healthy effects such as increased cardiac output, lockjaw and paresis of the extremities, dilatation of the vascular system, hypotension, and bradycardia (possibly leading to heart failure and cerebral haemorrhage)^6,15^. In addition, both have indirect toxic effects via their potentiating the toxicity of other BA, such as histamine. This occurs via the competitive inhibition of the detoxifying enzymes (diamine oxidase and histamine N-methyltransferase) involved in the oxidative catabolism of histamine^16–19^. The potentiation of histamine’s toxic effect may also be explained by putrescine and cadaverine facilitating the passage of histamine across the small intestine, thus increasing its rate of absorption into the blood stream^20,21^. In addition, putrescine and cadaverine can react with nitrites and produce nitrosamines (putrescine yields nitrosopyrrolidine and cadaverine nitrosopiperidine)^22^, compounds known to be carcinogenic^6,10,15^. Putrescine (which physiological concentration in the colonic lumen is normally in the milimolar range^23^) has also been indicated directly involved in the oncogenic process^24–26^. An association has also been reported between high intakes of dietary putrescine, along with the polyamines spermidine and spermine, and the risk of developing colorectal adenocarcinoma^26,27^.

Given these dangers, the legislation regarding the limits for BA in food needs revisiting. Indeed, in the European Union it has only been established a maximum legal limit for histamine^28^. Similarly, in the USA, the US Food and Drug Administration^29^ has only established legal limits for histamine in fish and fish products. No legislation has been established anywhere else, nor for any other BA. In fact, in a scientific opinion document regarding the risk-based control of BA formation in fermented products^12^, the EFSA panel on Biological Hazards (BIOHAZ) highlighted that a lack of knowledge prevented any reliable quantitative or qualitative risk assessment of putrescine and cadaverine in foods, concluding that further research on BA toxicity was needed.

Our group has recently developed an *in vitro* model for assessing the cytotoxicity of BA, either individually or in combination, in human intestinal cell cultures using real-time cell analysis (RTCA)^30,31^. With this model, tyramine and histamine were found to be cytotoxic towards intestinal cell cultures at concentrations easily reached in inherently BA-rich foods. In addition, it was revealed that while tyramine mainly causes cell necrosis, histamine induces apoptosis^31^. The model was also used to examine synergistic cytotoxicity between tyramine and histamine^30^. The aim of the present work was to examine, using the same model, the *in vitro* cytotoxicity of putrescine and cadaverine. The concentration of BA required to achieve half of the strongest cytotoxic effect observed in RTCA (IC_50_), the NOAEL, and the lowest observed adverse effect level (LOAEL), were calculated for both BA. The mode of action of each was also determined.

## Results

### Dynamic responses of putrescine and cadaverine-treated HT29 cells

**Figure 1:**
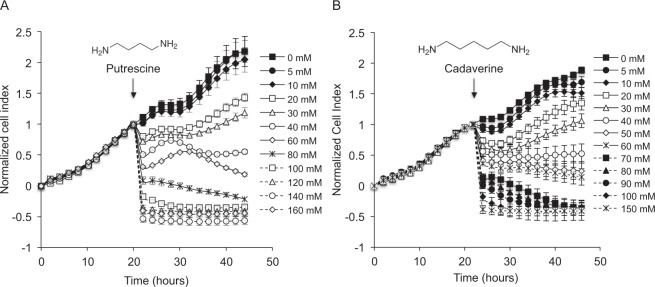
Real-time cell analysis of the effects of putrescine and cadaverine on HT29 cells. Cells were exposed to the indicated concentrations of putrescine (**A**), cadaverine (**B**) or control medium (0 mM). Vertical arrows show the point of administration of putrescine or cadaverine. The data correspond to a representative experiment of the triplicates achieved. Vertical bars represent standard deviations.

As determined by RTCA, a dose-dependent reduction in the normalized cell index was recorded for the HT29 cells exposed to putrescine (Fig. 1A) and cadaverine (Fig. 1B).

**Figure 2:**
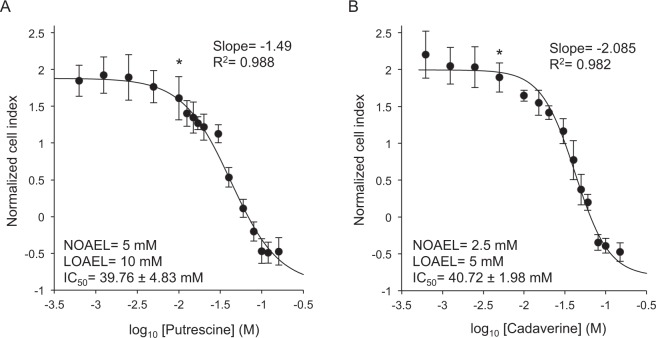
Dose-response curves for putrescine and cadaverine in HT29 intestinal cells. Cell cultures were exposed to a range of putrescine (**A**) or cadaverine (**B**) concentrations for 24 h. The curves were made by plotting the normalized cell index at 24 h of treatment obtained using RTCA, against the log_10_ value of the corresponding BA concentration. The data represent the means ± standard deviations of at least three independent experiments. An asterisk indicates the first significant difference with respect to the smallest dose of BA assayed (0.63 mM for both putrescine and cadaverine); it therefore corresponds to the LOAEL  (*p < 0.05). Numerical values for the IC_50_, NOAEL, LOAEL, Hill slope and R^2^ are also indicated.

The dose-response curves constructed after 24 h of putrescine (Fig. 2A) or cadaverine (Fig. 2B) exposure fitted a sigmoid curve with an R^2^ of 0.988 and 0.982 respectively. The Hill slope value for cadaverine (−2.085) was lower than that for putrescine (−1.49), indicating that for the same increase in concentration, a greater cytotoxic effect was seen with cadaverine than with putrescine.

To determine the cytotoxicity of putrescine and cadaverine towards HT29 intestinal cells, the IC_50_ values for each BA were determined at 8 h, 12 h, 18 h and 24 h (Table 1). The concentrations of putrescine and cadaverine required to cause half of the strongest cytotoxic effect observed in RTCA were progressively reduced with increasing incubation time. The IC_50_ values indicate that putrescine and cadaverine were similarly cytotoxic (IC_50_ for putrescine after 24 h of treatment 39.76 ± 4.83 mM, compared to 40.72 ± 1.98 mM for cadaverine). The NOAEL and LOAEL values for putrescine were 5 mM and 10 mM respectively, while for cadaverine they were 2.5 mM and 5 mM respectively (Fig. 2). The cytotoxicity threshold for cadaverine would therefore appear to be twice that of putrescine for HT29 cell cultures under the present experimental conditions.

**Table 1:** IC_50_ values (mean ± standard deviation) for putrescine and cadaverine determined after exposure of HT29 intestinal cells to these BA for different lengths of time.

Time	Putrescine	Cadaverine
(h)	(IC_50_)	(IC_50_)
8	112.28 ± 49.61	92.88 ± 44.17
12	91.89 ± 30.57	69.06 ± 18.55
18	46.40 ± 14.74	46.50 ± 4.94
24	39.76 ± 4.83	40.72 ± 1.98

Values are the mean ± standard deviation in mM.

### Microscopic examination of putrescine- and cadaverine-treated cell cultures

**Figure 3:**
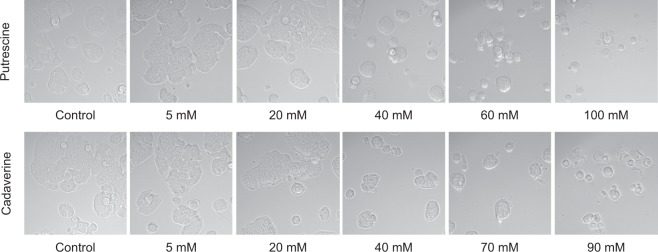
Toxicity towards HT29 cells of putrescine and cadaverine. HT29 cells were grown in flat-bottomed microplates for 20 h, and then exposed to increasing amounts of (**A**) putrescine (5, 20, 40, 60 and 100 mM) or (**B**) cadaverine (5, 20, 40, 70 and 90 mM) for 24 h. Cells were observed using an inverted optical microscope (magnification 40x).

Figure 3 shows the cytotoxic effects of different concentrations of putrescine and cadaverine on the HT29 cells. Concentrations for either BA below 20 mM had no effect on the morphology of the cells, or the number of cells. However, concentrations approximately above 20 mM caused an apparent progressive reduction in cell numbers and a gradual modification of cell morphology, confirming the dose-dependent cytotoxicity of both BA.

### Mode of action of putrescine and cadaverine

**Figure 4:**
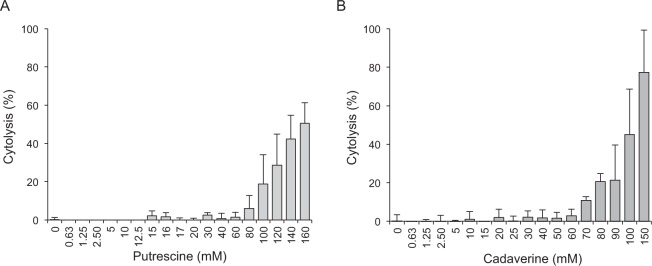
Determination of the cytolytic effect of putrescine (**A**) and cadaverine (**B**) on HT29 intestinal cells. RTCA cell culture supernatants were collected after 24 h of incubation with the corresponding concentration of putrescine or cadaverine. BA-induced necrosis was monitored as the release of cytosolic lactate dehydrogenase (LDH) into the culture medium. The data represent the percentage of cells lysed by treatment with different concentrations of putrescine or cadaverine. Data represent the means of at least three independent experiments; vertical bars represent standard deviations.

Neither putrescine nor cadaverine induced the formation of intracellular DNA fragments (a typical feature of apoptotic cells) in the cell cultures, either at their IC_50_ concentrations (0.97 ± 0.58% and 0.35 ± 0.43% DNA fragmentation for putrescine and cadaverine respectively) or at higher concentrations (3.6 ± 1.16% and 3.6 ± 1.27% DNA fragmentation for 80 mM putrescine and 70 mM cadaverine respectively) (no further data shown). Assays were therefore performed to measure the release of the cytosolic enzyme lactate dehydrogenase (LDH) into the medium to determine whether necrosis occurred. Figure 4A and B show the percentage cytolysis of the cell cultures exposed to increasing concentrations of putrescine and cadaverine respectively. Those exposed to putrescine concentrations below 80 mM, or cadaverine concentrations below 70 mM, showed negligible LDH activity. Above these concentrations, however, a dose-dependent increase in LDH activity was observed for both BA. Some 50.6% cytolysis was recorded in cultures exposed to the highest putrescine concentration tested (160 mM), and about 77.3% in cultures exposed to the highest cadaverine concentration tested (150 mM). Together, these results suggest putrescine and cadaverine have a necrotic rather than an apoptotic mode of cytotoxic action on these intestinal cells in culture.

## Discussion

The present results indicate putrescine and cadaverine to have a dose-dependent cytotoxic effect towards HT29 intestinal cells in culture. The cytotoxicity threshold for cadaverine was higher than that of putrescine; although their IC_50_ values were comparable, the NOAEL and LOAEL values for cadaverine were lower than those recorded for putrescine. To our knowledge, this is the first report of the IC_50_, NOAEL and LOAEL values for putrescine and cadaverine for *in vitro* cultures of an intestinal cell line. Recently, the values for these toxicological variables were RTCA-assessed for tyramine and histamine^31^. The comparison of the IC_50_, NOAEL and LOAEL values for putrescine and cadaverine with those recorded for tyramine and histamine^31^ indicates the former pair of BA to be less cytotoxic than the latter. Indeed, after 24 h of treatment, putrescine and cadaverine were approximately 10-times less cytotoxic to intestinal cells in culture than recorded for tyramine (IC_50_ putrescine 39.76 ± 4.83 and 40.72 ± 1.98 mM for cadaverine, compared to 3.2 ± 0.04 mM for tyramine^31^) and approximately 1.5 times less cytotoxic than histamine (IC_50_ histamine 26.0 ± 1.2 mM^31^). These data confirm and quantify the lesser toxicity assigned to these BA compared to histamine and tyramine, the most toxic of all dietary BA^12^.

Although the cytotoxic mode of action of putrescine and cadaverine was necrotic, as previously established for tyramine^31^, the percentage cytolysis caused by similar concentrations was much higher in tyramine-treated cells^31^. This further supports the lesser cytotoxicity of putrescine and cadaverine compared to tyramine. Further, while histamine caused the apoptosis of cell cultures^31^, negligible apoptotic DNA fragmentation was seen with putrescine and cadaverine. The necrotic effect of putrescine towards the HT29 intestinal cell cultures might be explained by its polycationic nature at physiological pH; this might allow it to interact with phospholipids (of anionic nature) and destabilize cell membranes^32,33^. A similar explanation might be proposed for the necrotic effect of cadaverine.

Our group recently reported the *in vitro* model used in this work to be helpful for estimating the risk of toxicity after the consumption of tyramine and histamine individually^31^ and in combination^30^. The present work also shows it to allow estimations for the risk of toxicity after the consumption of putrescine and cadaverine. The literature contains no human dose-response data for food-borne putrescine or cadaverine, and only one animal study has been performed^12,14^. Consequently, no legal limit has been established for putrescine in any food, even though this BA may accumulate at very high concentrations in cheese (up to 1560 mg/kg), fermented sausages (up to 1550 mg/kg), and fish sauces (up to 1220 mg/kg)^12^ - levels much higher than the lowest concentration of putrescine found to be cytotoxic in this work (LOAEL = 10 mM, equivalent to 881.50 mg/kg). These concentrations might therefore be considered hazardous to human health.

Similarly, no legal limit has been established for cadaverine in any food either^12^, even though it too may accumulate at very high concentrations in cheese (up to 3170 mg/kg), fish and fish products (up to 1690 mg/kg), fermented sausages (up to 1250 mg/kg) and fish sauces (up to 1150 mg/kg). In these foods, the cadaverine concentration reached can be much higher than the lowest concentration found to be cytotoxic in the present work (LOAEL = 5 mM, equivalent to 510.89 mg/kg). These concentrations might therefore also be considered hazardous for human health.

The high sensitivity of the *in vitro* model used in this study allowed to accurately estimate the NOAEL values for putrescine (5 mM, equivalent to 440.75 mg/kg) and cadaverine (2.5 mM, equivalent to 255.45 mg/kg), which were more than 4 and 7 times lower respectively than that obtained in oral toxicity studies performed in rats fed with diets containing putrescine or cadaverine, in which the NOAEL values for both BA were set at 2000 mg/kg^14^.

Given the possible potentiating effect of putrescine and cadaverine on the toxicity of other BA such as histamine and tyramine^16,19^, the threshold limits assigned for them should perhaps be even lower. Neither should it be forgotten that the toxicity of putrescine and cadaverine might be increased in certain consumers as a result of a reduced capacity to detoxify them (via a genetically or acquired impairment of amino oxidase activities). Children, elderly patients with gastrointestinal disease, and individuals taking monoamine and diamine oxidase inhibitors might also be at greater risk^6,10,12^.

The present results, plus those obtained in previous studies on tyramine and histamine^30,31^, suggest that tyramine is the most cytotoxic of these BA, followed by histamine, cadaverine, and finally putrescine.

## Conclusions

The results of this work show putrescine and cadaverine to be cytotoxic towards HT29 intestinal cell cultures at concentrations that can be easily reached in BA-rich foods. The IC_50_ value of putrescine was 39.76 ± 4.83 mM, while that of cadaverine was 40.72 ± 1.98 mM. The cytotoxicity threshold for cadaverine was twice that of putrescine; the LOAEL value for cadaverine was 5 mM while that for putrescine was 10 mM. These BA appear to exert their cytotoxic effects via the induction of necrosis rather than apoptosis. The present results may be useful to safety authorities in establishing legal limits for these BA in foods. This would help to prevent consumers suffering adverse health effects.

## Methods

### Cell line and growth conditions

The intestinal cell line HT29 (ECACC 91072201), derived from a human colorectal adenocarcinoma was used to provide an *in vitro* model of the intestinal epithelium. The cells were routinely cultured in McCoy’s 5a medium as described in Linares *et al*.^31^.

### Real-time cell analysis

An xCelligence Real-Time Cell Analyzer (ACEA Bioscience Inc., San Diego, CA, USA) was used as previously described^31^ to detect changes in the HT29 cells following their treatment with different concentrations of putrescine (1,4-diaminobutane dihydrochloride) (Sigma-Aldrich, Madrid, Spain) or cadaverine (1,5-diaminopentane dihydrochloride) (Sigma-Aldrich).

In short, HT29 cells were seeded at a density of 2 × 10^4^ cells/well in 16-well E-Plates (ACEA Biosciences Inc.) containing 100 µl of medium per well, and then incubated and monitored in a Heracell-240 Incubator (Thermo Electron LDD GmbH, Langenselbold, Germany) at 37 °C under a 5% CO_2_ atmosphere^31^. Stock solutions of putrescine and cadaverine were dissolved in water and adjusted to pH 6.9. Approximately 20 h after seeding, the cells were treated with one of 15 concentrations of putrescine (0 to 160 mM) or cadaverine (0 to 150 mM). The final volume of culture media supplemented with BA was 200 µl per well. The cell index was monitored for 24 h; this was normalized to the time point just previously the addition of the BA and set to 1. For each condition, measurements were done in triplicate.

Dose-response curves for putrescine and cadaverine were made by plotting the normalized cell index at 24 h of treatment (obtained using RTCA software 1.2.1; ACEA Biosciences Inc.) against the log_10_ value of the corresponding BA concentration (0.63, 1.25, 2.50, 5, 10, 12.5, 15, 17, 20, 30, 40, 60, 80, 100, 120 and 160 mM for putrescine and 0.63, 1.25, 2.50, 5, 10, 15, 20, 25, 30, 40, 50, 60, 70, 80, 90, 100 and 150 mM for cadaverine). Non-linear regression trend lines were fitted to sigmoid dose-response (variable slope) curves using SigmaPlot 13.0 software (Systat Software Inc., San Jose, CA, USA). This software was also used to determine the coefficient of determination (R^2^), which indicates the goodness of the adjustment of the experimental data to the curve, as well as the Hill slope value, which shows the steepness of the curve.

The IC_50_ values for putrescine and cadaverine were calculated at different arbitrary time points (8 h, 12 h, 18 h and 24 h) of BA exposure as described in Linares *et al*.^31^.

### Live cell microscopy

Cells were seeded at a density of 2 × 10^4^ cells/well and incubated in flat-bottomed 96-well microplates under the same conditions to those used in the RTCA studies. After 24 h of incubation, the cells were treated with concentrations of putrescine (5, 20, 40, 60 and 100 mM) and cadaverine (5, 20, 40, 70, 90 mM). At 24 h post-treatment, live cells were observed using an inverted LumaScope-600 Series optical microscope (Etaluma, Carlsbad, CA, USA) with a 40x objective.

### Cell apoptosis

The Cellular DNA Fragmentation ELISA Kit (Roche Applied Science, Germany) was used to measure apoptosis-associated DNA fragments in the cytoplasm. DNA-fragmentation in BA-treated samples was determined as described in Linares *et al*.^31^ with some modifications [proliferating cells were exposed for 24 h to BA doses similar to the IC_50_ (35.48 mM for putrescine and 41.03 mM for cadaverine), and above the IC_50_ (80 mM for putrescine and 70 mM for cadaverine)]. Positive and negative controls were performed as described in Linares *et al*.^31^. DNA fragmentation in the BA-treated samples was indicated as a percentage of the value for the positive controls.

### Cell lysis assay

The presence of LDH activity in RTCA cell culture supernatants collected after 24 h of incubation with the corresponding dose of putrescine or cadaverine, was tested as an indicator of cell lysis, using the Cytoscan Cytotoxicity Assay Kit (G Biosciences, St. Louis, MO, USA) following the manufacturer’s instructions. Negative (no lysis reagent) and positive (with lysis reagent) controls were performed simultaneously. The percentage of cells lysed was determined as follows: 100 × [(492 nm absorbance of BA-treated samples − absorbance of negative control)/(absorbance of positive control − absorbance of negative control)].

### Data and statistical analysis

The results of the different experiments were indicated as the mean ± standard deviation of three independent replicates. Statistical treatment involved ANOVA followed by Fisher’s least significant difference test, performed using SigmaPlot software. Significance was set at *p* < 0.05 (indicated in figures with an asterisk).

The NOAEL value was identified as the highest concentration of BA that caused no detectable adverse effect on the target cells; the LOAEL value was defined as the lowest concentration of BA that produced a detectable adverse effect^31^.
